# Case report: Efgartigimod is a novel therapeutic option for ocular myasthenia gravis: a report of 2 cases

**DOI:** 10.3389/fimmu.2024.1497398

**Published:** 2025-01-13

**Authors:** Tianying Ma, Ying Zhu, Ruixia Zhu

**Affiliations:** Department of Neurology, The First Affiliated Hospital of China Medical University, Shenyang, China

**Keywords:** efgartigimod, ocular myasthenia gravis, case report, AChR-ab, FcRn

## Abstract

**Introduction:**

Efgartigimod has been approved for the treatment of acetylcholine receptor antibodies-positive generalized myasthenia gravis (AChR-Ab+gMG), but its efficacy in patients with ocular myasthenia gravis (OMG) is not known.

**Case presentation:**

We describe 2 cases of patients with AChR-Ab+ OMG who showed unfavorable responses to corticosteroids and tacrolimus. Within 2 weeks of initiating efgartigimod, both patients showed rapid improvement and minimal symptom expression was achieved in weeks 3 to 4, which was maintained up to week 12.

**Conclusion:**

The 2 cases described herein provide preliminary evidence for the effectiveness of efgartigimod for the treatment of OMG for patients who do not respond or are intolerant to conventional medications. Large-scale studies are needed to confirm these findings.

## Introduction

Myasthenia gravis (MG) is an autoimmune disease affecting the neuromuscular junction; it is primarily caused by the production of anti-acetylcholine receptor antibodies (AChR-Ab) that induce progressive muscle weakness. Some patients produce antibodies against muscle-specific tyrosine kinase (MuSK) or low-density lipoprotein receptor-related protein4 (LRP-4) whereas others do not, and up to 85% of patients present with extraocular muscle weakness at disease onset. Ocular MG (OMG) accounts for 50% of all MG cases in China and approximately 80% of these progress to generalized myasthenia gravis (gMG) within 2 years, highlighting the importance of early intervention. International consensus guidelines recommend corticosteroids for patients who do not show a satisfactory response to pyridostigmine (1). However, in some cases symptoms deteriorate with corticosteroid treatment, which negatively impacts patients’ daily activities and quality of life. Therefore, there is an urgent need for more effective treatments to alleviate extraocular muscle weakness in patients with MG. Here we describe 2 cases of OMG that were successfully treated with efgartigimod, a first-in-class humanized IgG1 Fc fragment that was approved by the United States Food and Drug Administration for the treatment of AChR-Ab+ gMG.

## Case 1

**Figure 1:**
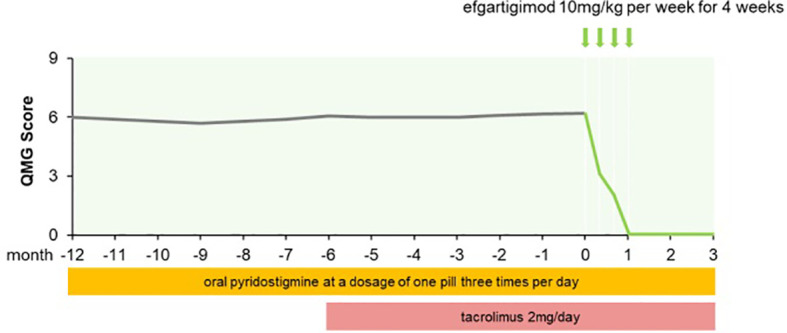
QMG score changes of patient 1 from MG onset to follow-up for three months after afgartigimod.

A 71-year-old man with a 1-year history of fluctuating right eyelid ptosis and diplopia was admitted to our hospital. The results of the neostigmine test were positive. Serum AChR-Ab titer was 1.25 nmol/L. No thymoma was detected by chest computed tomography (CT). The patient was ultimately diagnosed with OMG. He was initially treated with oral pyridostigmine (1 tablet 3 times daily) but did not show a satisfactory response: he continued to exhibit impaired vision, diplopia, and ptosis. Tacrolimus (2 mg/day) was administered; after 2 months, the dosage was adjusted to achieve a blood tacrolimus concentration of 8.5 ng/mL. However, the ocular symptoms remained unresolved, which greatly impacted the patient’s quality of life as he was unable to leave his home and perform activities independently, requiring the support of his wife. Because of concerns regarding the side effects of corticosteroids, the patient refused prednisone and instead received efgartigimod (10 mg/kg per week for 4 weeks). After the second infusion, the patient showed improvements in visual clarity and the amplitude of eyelid elevation; his Quantitative Myasthenia Gravis (QMG) score was 3. After the third infusion his score was 2 and after the fourth infusion there was complete resolution of ocular symptoms, with a QMG score of 0 (**Figure 1**). The patient’s AChR-Ab titer has decreased to 0.78 nmol/L after 1 month and he maintains minimal symptom expression (MSE) status as of this writing.

## Case 2

**Figure 2:**
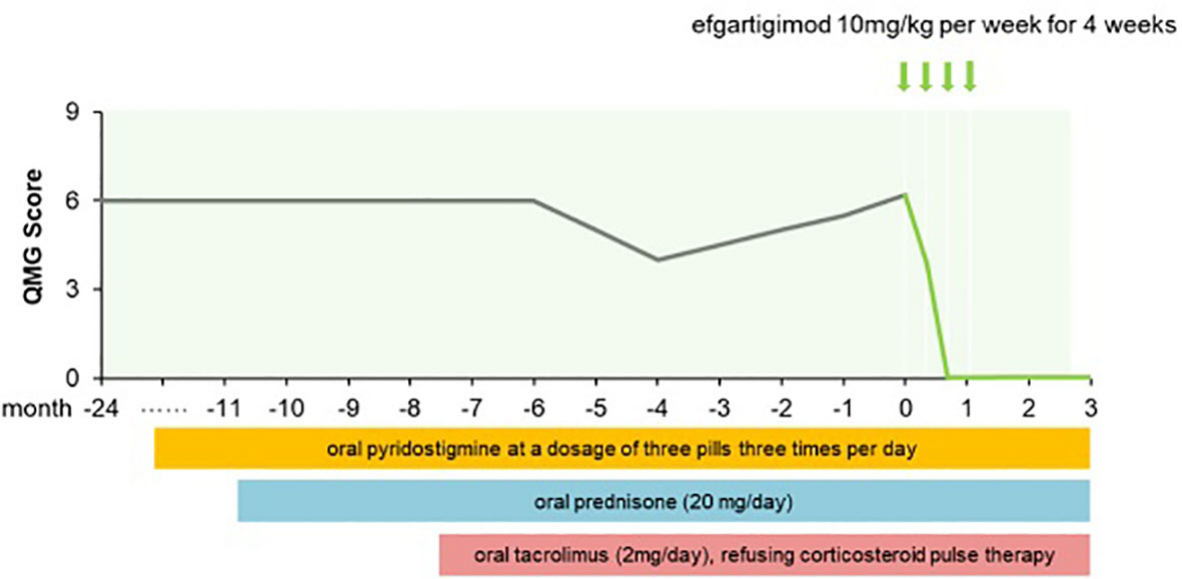
QMG score changes of patient 2 from MG onset to follow-up for three months after afgartigimod.

Two years ago, a 64-year-old man presented at our hospital with left eyelid ptosis and visual field deficits. Several days later he developed bilateral eyelid ptosis and diplopia that fluctuated over the course of the day, with an alleviation of symptoms in the morning and a worsening in the evening. The patient was positive for the neostigmine test and AChR-Ab titer was 5.44 nmol/L. Electromyography revealed a low-frequency decremental response of the left facial nerve. No thymoma was detected on a chest CT scan. Based on his symptoms and the results of examinations, the patient was diagnosed with OMG. He was given oral pyridostigmine (3 tablets daily) but there was no notable symptom improvement. Oral prednisolone (20 mg/day) and oral tacrolimus (2 mg/day) were then administered and target blood tacrolimus concentration was revised to 2.4–4 ng/mL after 2 months. The patient’s blood glucose level was elevated, prompting the administration of oral hypoglycemic drugs. Despite these interventions, the patient’s eyelid ptosis and diplopia persisted, which negatively impacted his quality of life: he was unable to leave his home or engage in activities such as driving or attending a class reunion for fear of being mocked. He refused further corticosteroid pulse therapy and did not respond to tarolimus and low-dose prednisone. Efgartigimod was initiated as an add-on treatment. After the initial infusion, the patient showed improvements in visual clarity and the amplitude of eyelid elevation, with a QMG score of 4. After the third infusion his ocular symptoms were fully resolved, with a QMG score of 0 (**Figure 2**). AChR-Ab titer was 1.72 nmol/L. At week 12 he continued to maintain MSE status.

## Discussion

We report 2 cases of elderly patients with OMG with no obvious improvement after using prednisone and tacrolimus. After switching to efgartigimod for 1 month, the patients showed complete resolution of their symptoms. After 3 months of follow up, the patients’ symptoms remained stable. Moreover, no adverse drug reactions were found in our two patients. There are 2 possible explanations for the large proportion of patients with MG (85%) presenting with extraocular muscle weakness at disease onset (2). First, it has been demonstrated that both fetal and mature AChR types (2αβγδ and 2αβϵδ, respectively) are expressed in mature extraocular muscles. The γ subunit contributes to the development of ocular symptoms by inducing an autoimmune response against fetal-type AChR in extraocular muscle: pathogenic AChR-Abs attack AChR expressed in the postsynaptic membrane of the neuromuscular junction. Additionally, extraocular muscle twitch fibers tend to block neuromuscular transmission with their high firing frequency and lower AChR density and ACh release rate at synapses. Whereas Efgartigimod is a human IgG1 antibody Fc fragment that binds to FcRn and competitively inhibits the binding of endogenous IgG to FcRn, promoting the degradation of IgG in lysosomes and thus accelerating the clearance of pathogenic IgG in the body (3).We speculate that efgartigimod improved OMG symptoms in our patients by decreasing the level of pathogenic AChR-Ab, thereby alleviating the destruction of the extraocular muscle neuromuscular junction and preserving its normal structure.

Oral prednisone is commonly prescribed to patients with OMG. However, the therapeutic effect is inadequate in some cases, necessitating a treatment switch. Intravenous methylprednisolone (IVMP) and tacrolimus monotherapy are recommended for patients who show a poor response to oral prednisone (4). However, in clinical practice, the treatment algorithm for OMG depends on a number of factors such as disease severity, the presence of contraindications, and patient preference. Corticosteroid pulse therapy is associated with adverse effects including osteoporosis, hypertension, exacerbation of diabetes mellitus, gastrointestinal ulcer, cataract, and opportunistic infection. Furthermore, many older patients have multiple comorbidities that preclude the use of IVMP. It is noted that older patients often have some comorbidities such as diabetes mellitus, hypertension and hyperlipidemia which would be aggravated after the administration of corticosteroid and tacrolimus. Moreover, some elderly patients may take medications such as statins and beta-blockers for comorbidities that are contraindicated for MG treatment. Based on these, clinicians need to draw up a personalized safe medication regimen for older patients and monitor serum biochemical index (5). The cases described herein provide evidence that efgartigimod is an effective therapeutic option for alleviating ocular symptoms associated with MG and is also better safety.

Thymoma, elevated AChR-Ab titers, and a decremental response to low-frequency repetitive nerve stimulation are risk factors for the evolution of OMG to gMG (6). Immunosuppression not only improves ocular symptoms in patients with OMG but can also prevent the progression to gMG (7). Therefore, patients with OMG should be thoroughly evaluated to guide the selection of an appropriate and effective treatment.

Although efgartigimod has been approved for the treatment of AChR Ab+ systemic MG, an analysis of the ocular muscle subgroup from the ADAPT phase 3 trial showed that in week 2 of the first treatment cycle with efgartigimod, MG Activities of Daily Living (MG-ADL) scale score was significantly improved compared with the placebo group (−0.82 vs. −0.18, p=0.009); and there was further improvement by week 4 of the second cycle (−1.49 vs. −0.25, p<0.001) (3). These data demonstrate that efgartigimod can mitigate ocular symptoms in patients with MG.

In conclusion, our findings provide preliminary real-world evidence for the effectiveness of efgartigimod as a novel treatment option for patients with OMG who fail to respond or are intolerant to conventional medications. However, a large-scale study is needed to confirm these findings.

## Data Availability

The original contributions presented in the study are included in the article/supplementary material. Further inquiries can be directed to the corresponding author.
